# Nucleophosmin 1 proteins as potential therapeutic targets in non-communicable chronic inflammatory diseases: a review of pathophysiological mechanisms

**DOI:** 10.3389/fcell.2026.1840902

**Published:** 2026-07-08

**Authors:** Kaijie Wen, Tao Hu, Qiang Wang, Xiongshan Sun

**Affiliations:** 1 Department of Clinical Medicine, North Sichuan Medical College, Nanchong, Sichuan, China; 2 Department of Cardiovascular Medicine, The General Hospital of Western Theater Command, Chengdu, China

**Keywords:** autophagy, cell differentiation, chronic inflammatory diseases, epigenetics, nucleolar stress, nucleophosmin

## Abstract

Nucleophosmin1 (NPM1) proteins, initially recognized as central guardians of nucleolar architecture and function, have recently been redefined as pivotal hubs that integrate diverse forms of chronic cellular stress signaling. Although the roles of NPM1 have been extensively elucidated in tumor biology, its broad involvement in non-communicable chronic inflammatory diseases (NCDs) remains insufficiently and unsystematically summarized. Here, we highlight NPM1 as a key sensor of stress-induced nucleolar disassembly, nucleocytoplasmic translocation, and p53 stabilization. In pathological conditions such as myocardial ischemia, endothelial dysfunction, atherosclerosis, and chemotherapy-associated cardiotoxicity, NPM1 exhibits pronounced context dependence functioning either to initiate cytoprotective responses or to promote inflammation and apoptosis. In parallel, NPM1 plays a central role in maintaining genomic stability by sequestering, mobilizing, and regulating essential enzymes across multiple DNA damage repair pathways, including base excision repair (BER) and translesion synthesis (TLS). Dysregulation of these functions is closely linked to chronic pathological processes driven by metabolic stress, oxidative stress, and proteotoxicity. Collectively, available evidence suggests that NPM1, as a core node of the nucleolus–nucleoplasm signaling axis, may constitute a common molecular pathological basis underlying multiple chronic inflammatory diseases, including cancer, cardiovascular diseases, diabetes, and neurodegenerative disorders. A deeper dissection of its post-translational modifications, stress-dependent subcellular re-localization, and interactions with partner proteins is expected to provide a novel conceptual framework and therapeutic avenues for the development of NPM1-based targeted interventions. Accordingly, this review synthesizes the core molecular mechanisms of the NPM1 in the maintenance of cellular homeostasis, including regulating nucleolar stress, DNA damage repair, and inflammation, We place a particular emphasis on how these baseline pathways translate into distinct functional phenotypes within the pathological processes of chronic diseases, including cardiovascular, metabolic, and neurodegenerative disorders.

## 1 Introduction

Non-communicable chronic inflammatory diseases (NCDs), including cancers, metabolic disorders, neurodegenerative diseases, and chronic cardiovascular diseases, have emerged as the predominant threats to global health. Although these diseases exhibit highly diverse clinical phenotypes, they often share common pathophysiological basis. Specifically, when cells are subjected to persistent genotoxic stress, metabolic dysregulation, oxidative stress, and proteotoxic stress, the intrinsic mechanisms that maintain cellular homeostasis will gradually collapse. Consequently, elucidating how cells sense and respond to chronic stress signals is crucial to reveal the potential mechanisms underlying these diseases and to develop broad-spectrum therapeutic strategies.

Among the cellular stress sensors, the nucleolus is undergoing a role revolution. Rather than serving merely as a ribosome factory, the nucleolus is now recognized as a highly dynamic hub for the integration of cellular stress signals. Nucleolar stress, triggered by impaired ribosome biogenesis, is increasingly regarded as a key contributor to the pathophysiology of multiple chronic inflammatory diseases (Yan and Hua, 2022). Within this stress-response network, NPM1 occupies a central position. As a highly abundant and functionally diverse nucleolar protein, NPM1 shuttles across the nucleolus, nucleoplasm, cytoplasm, and even the extracellular space (Box et al., 2016). NPM1’s functions encompass nearly all core life processes, including ribosome biogenesis (Lindström, 2011), histone chaperoning (Lindström, 2011), DNA damage repair (López et al., 2020a), and the maintenance of genomic stability (Taha and Ahmadian, 2024).

However, there is a great disparity in the current research landscape of NPM1. On one hand, its role in tumor biology, especially in acute myeloid leukemia (AML) has been elucidated in exceptional depth (Hindley et al., 2021). Pathogenic mechanisms driven by NPM1 mutation (NPM1c+), including epigenetic remodeling, differentiation blockade, and evasion of apoptosis, have established NPM1 as one of the most critical diagnostic markers and therapeutic targets in AML (Sharma and Liesveld, 2023). These studies have provided a rich basis for understanding the molecular regulation of NPM1.

On the other hand, research on the role of the NPM1 in other NCDs, such as neurodegenerative disorders, cardiovascular diseases, and metabolic dysfunction, still remains original, scattered, and fragmented. Does NPM1 play roles in neurons analogous to those it assumes in cancer cells? (Kreiner et al., 2013) Is NPM1 a guardian or a perpetrator in cardiomyocytes? (Yan and Hua, 2022) Do its finely tuned epigenetic regulatory mechanisms also mediate post–myocardial infarction inflammatory responses? (Zhang et al., 2024) Does NPM1 play roles in endothelial dysfunction (Kinumi et al., 2005), atherosclerotic plaque formation (Rao et al., 2021), and the progression of diabetes (Tang et al., 2025)? This review seeks to systematically address these emerging questions by mapping NPM1’s functional interactome across various NCDs.

Accordingly, this review aims to systematically synthesize the core molecular mechanisms of NPM1 proteins under cellular stress. Simultaneously, we integratively link these baseline mechanisms to the complex functional phenotypes that drive the pathological progression of various NCDs, including cardiovascular disorders and metabolic dysregulation. Thus, we seek to highlight the universal significance of NPM1 as a central regulatory node and to provide novel perspectives and potential therapeutic targets for the treatment of chronic inflammatory diseases. To achieve this, we conducted a comprehensive literature search in PubMed and Web of Science using keywords including “Nucleophosmin,” “NPM1,” “cardiovascular diseases,” “metabolic disorders,” and “neurodegenerative diseases” to summarize the latest evidence.

## 2 Core mechanisms of NPM1 in cellular homeostasis and stress response

### 2.1 Regulation of nucleolar stress

**FIGURE 1:**
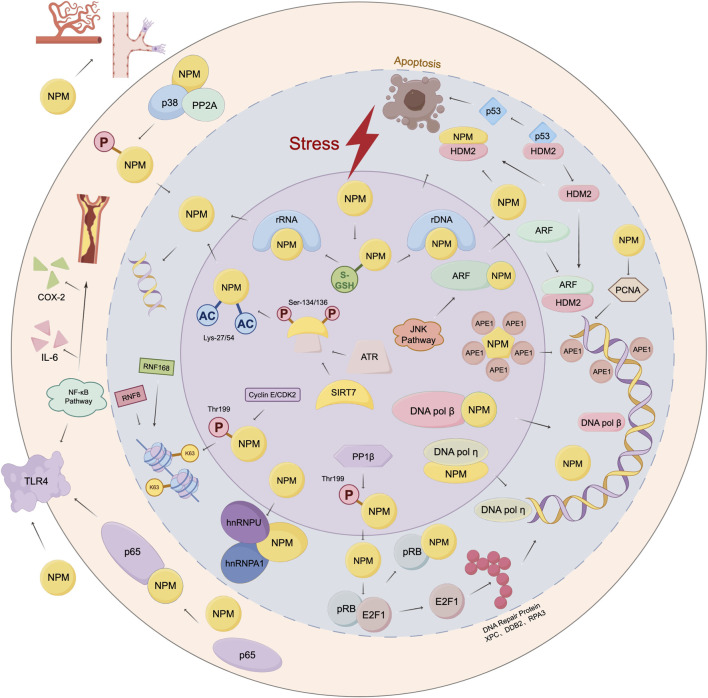
NPM1 acts as a dynamic nucleolar-nucleoplasmic-cytoplasmic stress sensor. In the resting state, NPM1 predominantly exists as a pentamer within the nucleolus, where it anchors key proteins such as p14ARF and APE1. Under stress conditions, NPM1 undergoes post-translational modifications and translocates to the nucleoplasm, cytoplasm, and even the extracellular space. Nucleoplasmic NPM1 releases p14ARF to activate the p53 pathway and recruits repair factors such as APE1 to participate in DDR. Cytoplasmic NPM1 either drives inflammatory signaling by interacting with NF-κB or is secreted extracellularly to act as an alarmin activating TLR4 signaling. Crucially, S-glutathionylation at Cys275 and dephosphorylation at Thr199 are critical stress-induced modifications that trigger NPM1’s dissociation from the nucleolus and its functional switch in the nucleoplasm/cytoplasm. This stress-induced dynamic relocalization enables NPM1 to regulate cell fate in a multidimensional and context-dependent manner.

Nucleolar stress, a cellular stress response triggered by impaired ribosome biogenesis, is increasingly recognized as a key contributor to the pathophysiology of multiple NCDs, including neurodegenerative disorders and cardiovascular diseases (Yan and Hua, 2022; Marquez-Lona et al., 2012). A variety of chronic adverse stimuli, such as ischemia/reperfusion, hypoxia, oxidative stress, DNA damage, or exposure to chemotherapeutic agents, can induce nucleolar stress, which is typically accompanied by the redistribution and functional remodeling of core nucleolar proteins (Figure 1) (Sengar et al., 2024).

In cardiomyocytes, nucleolar stress represents an early response to myocardial injury (Sengar et al., 2024) and exerts pleiotropic effects on cardiac repair. For instance, during myocardial infarction, nucleolar stress in the acute injury phase promotes cardiomyocyte survival and repair through the upregulation of nucleolar proteins such as NPM1, nucleostemin, and nucleolin. Clinically, patients with severe cardiac disease exhibit a reduction in AgNOR regions together with enlarged nucleoli, suggesting that chronic nucleolar dysfunction occurs in parallel with cardiac aging and functional dysfunction (Yan and Hua, 2022). Another study has also confirmed persistent features of nucleolar stress in cardiac tissues from patients with ischemic or dilated cardiomyopathy. These features include nucleolar enlargement, increased fibrillar centers, perinucleolar chromatin, and dense fibrillar components, along with marked upregulation of nucleolar proteins. Although no obvious changes in NPM1 levels were observed in that study, the structural alterations of the nucleolus strongly imply its potential involvement (Roselló et al., 2012). A recent study has demonstrated that in the myocardial nuclei from patients undergoing cardiac surgery who suffer from other chronic inflammatory diseases, nucleolin occupies a significantly larger area of the nucleus, suggesting possible alterations in nucleolar structure and potential nucleolar stress (Tomkova et al., 2024). Notably, nucleolar stress can also directly drive cardiomyocyte death, a phenomenon that is particularly evident in chemotherapy-induced cardiotoxicity (Sengar et al., 2024).

Nucleolar stress also plays important roles in other cardiovascular cell types. In vascular smooth muscle cells, nucleolar stress activates the ATM/ATR/p53 axis through the DNA damage response, leading to extensive p53 phosphorylation, the induction of a senescence-like phenotype, and the development of degenerative vascular diseases such as abdominal aortic aneurysm (Zhang et al., 2020). In vascular endothelial cells, nucleolar stress induced by split end inhibition, which leads to pRNA upregulation and subsequent suppression of 47S pre-rRNA synthesis, suppresses cell proliferation via p53-dependent signaling and impairs pathological angiogenesis characterized by high permeability and low perfusion efficiency (Yang et al., 2023). Collectively, these findings underscore the complexity of nucleolar stress in the cardiovascular system.

Pathological processes initiated by diverse stressors critically depend on the precise regulation of sensor proteins that respond to changes in nucleolar homeostasis. Among the proteins responsive to alterations in nucleolar homeostasis, NPM1 has been identified as a core sensor mediating nucleolar stress signaling pathways (Taha and Ahmadian, 2024; Yao et al., 2010). Under various cellular stress, including ultraviolet irradiation or oxidative stress, NPM1 undergoes dynamic subcellular redistribution, a hallmark event of nucleolar stress (Taha and Ahmadian, 2024; Yang et al., 2016). The central mechanism underlying this process involves changes in the intranucleolar redox state. Stress-induced nucleolar oxidation leads to S-glutathionylation of NPM1 at Cys275, a modification that disrupts its interaction with nucleolar nucleic acids such as rDNA and rRNA, thereby promoting the dissociation of NPM1 from the nucleolus and translocation into the nucleoplasm (Yang et al., 2016). Another study demonstrated that under genotoxic stress, activation of PP1β results in dephosphorylation of NPM1 at Thr199, a residue previously phosphorylated by TPL2, thereby facilitating NPM1 nuclear export (Eliopoulos and Volarevic, 2015).

Once entering the nucleoplasm, NPM1 directly interacts with HDM2, the E3 ubiquitin ligase of p53, thereby inhibiting HDM2-mediated ubiquitination and degradation of p53. The effective sequestration of HDM2 ultimately stabilizes and accumulates p53, activates its transcriptional activity, and initiates downstream stress-response programs, including cell cycle arrest and apoptosis (Yang et al., 2016; Eliopoulos and Volarevic, 2015; Kurki et al., 2004). Intriguingly, in the context of myocardial injury, the release of nucleolar proteins into the nucleoplasm appears to stabilize HDM2, thereby restraining excessive p53 activation and limiting apoptosis (Yan and Hua, 2022). In addition, under nucleolar stress, hnRNPU is upregulated, and NPM1 sequentially interacts with hnRNPU and hnRNPA1 to form a ternary complex that promotes cell survival (Yao et al., 2010). In neuronal cells, nucleolar stress is associated with downregulation of NPM1 concomitant with p53 upregulation. Additionally, NPM1 can directly activate the mitochondrial apoptotic pathway through a mechanism that is partially independent of the canonical p53 signaling cascade (Marquez-Lona et al., 2012). In endometrial stromal cells, nucleolar stress-induced NPM1 translocation has been shown to promote mesenchymal-to-epithelial transition (MET) during decidualization, while simultaneously suppressing the expression of differentiation markers, suggesting that excessive nucleolar stress is detrimental to induce cellular differentiation (Liang et al., 2020).

NPM1-mediated nucleolar stress signaling exhibits pronounced contextual specificity within the cardiovascular system (Hariharan and Sussman, 2014). During the early stage of myocardial injury such as hypoxia, ischemia/reperfusion, or doxorubicin (Dox)-induced cardiotoxicity, upregulation of NPM1 expression and its translocation from the nucleolus to the nucleoplasm represent a common early stress response in cardiomyocytes and cardiac progenitor cells (Avitabile et al., 2011). This translocation stabilizes p53 and initiates cytoprotective programs with anti-apoptotic effects, thereby safeguarding cardiomyocytes from cell death during acute stress (Avitabile et al., 2011). In contrast, during chronic pathological processes such as cardiac hypertrophy and the recovery phase post-myocardial infarction, NPM1 can compensatorily enhance nucleolar function to meet the increased demand for protein synthesis, thus sustaining cellular survival (Avitabile et al., 2011). Nevertheless, the function of NPM1 is not uniformly protective. In human umbilical vein endothelial cells (HUVECs), oxidative stress induces dephosphorylation of NPM1 at Thr199 and its translocation from the cytoplasm to the nucleus, where it interferes with DNA damage repair (DDR) signaling pathways, revealing a potential detrimental role of NPM1 in the regulation of endothelial dysfunction (Guillonneau et al., 2016).

Another critical function of NPM1 under nucleolar stress is its regulation of inflammation, which is mediated by its distinct subcellular localizations. Under stress stimuli, NPM1 is secreted into the extracellular space, where it acts as an alarmin. By interacting with toll-like receptor 4 (TLR4) on the cell membrane, extracellular NPM1 activates the NF-κB inflammatory pathway and induces the expression of pro-inflammatory mediators such as interleukin-6 (IL-6) and cyclooxygenase-2 (COX-2), thereby exacerbating vascular inflammation (Beji et al., 2021). Another study has shown that stress-induced extracellular NPM1 released from endothelial cells also promotes vascular inflammation through upregulation of intercellular adhesion molecule-1 (ICAM-1) (Di Carlo et al., 2021).

Additionally, NPM1 can also directly drive inflammatory signaling within cells. This role is particularly prominent in atherosclerosis, a prototypical chronic inflammatory disease. Early studies have reported that upon stimulation with oxidized low-density lipoprotein (oxLDL), NPM1, normally a constitutive component of HUVECs (Bruneel et al., 2003), undergoes extensive dephosphorylation (Kinumi et al., 2005). More recently, Caijun Rao and colleagues also demonstrated that NPM1 is markedly upregulated in endothelial cells within human atherosclerotic plaques. Mechanistically, cytoplasmic NPM1 directly binds to the p65 subunit of NF-κB and promotes its phosphorylation, thereby activating NF-κB signaling and ultimately aggravating endothelial inflammation and dysfunction (Rao et al., 2021; Csiszar et al., 2008). These stress-induced changes in NPM1 localization and function are also modulated by other signaling pathways. For example, under endothelial stress conditions, heme oxygenase-1 (HO-1) can be cleaved by signal peptide peptidase (SPP) and translocates from the endoplasmic reticulum to the nucleus, where it binds to the N-terminal region of NPM1. This interaction physically masks the nuclear export signal (NES) of NPM1, thereby inhibiting its nucleocytoplasmic shuttling and blocking NPM1-mediated p53 activation, ultimately protecting against endothelial cell senescence (Luo et al., 2021).

Together, these findings highlight NPM1 as a generalized nucleolar stress-response hub that exerts pleiotropic roles across different cell types and pathological stages in chronic inflammatory diseases such as atherosclerosis, neurodegenerative disorders. Targeting specific post-translational modifications, subcellular localization, or molecular partners of NPM1 may offer novel methods for the precise treatment of chronic stress-related diseases.

### 2.2 Regulation of DNA repair

NPM1 is primarily associated with multiple cellular processes, including ribosome biogenesis, cell cycle progression, apoptosis, and cell differentiation (Lin et al., 2010). However, accumulating evidence indicates that NPM1 also plays a central role in the maintenance of genome stability (Poletto et al., 2014; Scott and Oeffinger, 2016). For example, knockdown of NPM1 in embryonic cells increases the presence of γ-H2AX, the double-strand break marker (Colombo et al., 2005), suggesting that NPM1 is not only as a sensor and responder to DNA damage stress (Scott and Oeffinger, 2016; Yogev et al., 2008), but also participates in multiple DDR pathways (Scott and Oeffinger, 2016).

Under normal physiological conditions, the RNA-binding activity of NPM1 remains consistently low (Yang et al., 2002). Leveraging its high abundance in the nucleolus and its multi-domain structure, NPM1 sequesters various key DDR proteins within the nucleolus, thereby limiting their functional activity in the nucleoplasm. A canonical example is NPM1’s binding to the tumor suppressor p14ARF (p19ARF in mouse) to the nucleolus via its N-terminal domain (Colombo et al., 2005; Yogev et al., 2008; Lee et al., 2005; Gjerset, 2006), which prevents p14ARF from interacting with the E3 ubiquitin ligase HDM2 (Mdm2 in mouse) in the nucleoplasm, thereby ensuring the normal degradation of the p53 protein and maintaining its low intracellular levels (Taha and Ahmadian, 2024; Lee et al., 2005). Concurrently, NPM1 anchors key BER enzymes, such as APE1 and FEN1, as well as critical proteins from the TLS pathway, including DNA polymerase η, within the nucleolus to protect them from proteasomal degradation (López et al., 2020a; Poletto et al., 2014; Scott and Oeffinger, 2016; Ziv et al., 2014; López et al., 2020b). NPM1 is likewise regulated by upstream factors. The phosphatase DUSP3 constitutively binds to NPM1 and keeps tyrosine phosphorylation at Y29, Y67, and Y271 at a low state, thereby maintaining NPM1’s nucleolar anchoring and genomic stability (Russo et al., 2021).

Under stress conditions, NPM1 activates its RNA-binding activity through post-translational modifications (PTMs), enabling it to specifically bind to hairpin-structured RNA (Yang et al., 2002). Subsequently, NPM1 is released into the nucleoplasm through several pathways. Dissociation of the ARF-NPM1 complex liberates both ARF and NPM1 to the nucleoplasm (Lee et al., 2005; Gjerset, 2006). Moreover, activation of ATR during genotoxic stress promotes its interaction with SIRT7 and phosphorylation of SIRT7 at Ser134/136. Activated SIRT7 subsequently deacetylates NPM1 at Lys27 and Lys54, facilitating the release of NPM1 to the nucleolus (Ianni et al., 2021). Both ARF and NPM1 bind to MDM2 to inhibit its function, leading to p53 upregulation (Lee et al., 2005; Gjerset, 2006). This process is tightly controlled by upstream signaling cascades. Activation of the JNK pathway and its downstream effectors, c-Jun and JunB, serve as critical drivers mediating the DNA damage-dependent nucleolar-to-nucleoplasmic translocation of NPM1 and ARF. Phosphorylation of c-Jun at Thr91/93 promotes its binding to NPM1 and pulling NPM1 out of the nucleolus, thereby facilitating the dissociation of the ARF-NPM1 complex (Yogev et al., 2008).

Accompanied by the release of NPM1, various key enzymes from DNA repair pathways are also translocated to the nucleoplasm, where they collaborate with NPM1 to play a crucial role in DNA repair (Poletto et al., 2014). Leveraging its unique pentameric structure, NPM1 binds several APE1 molecules and transports them to DNA damage sites, while simultaneously enhancing the specific binding of APE1 to DNA containing genuine apurinic/apyrimidinic (AP) sites, thereby improving the efficiency of DNA repair (López et al., 2020b). NPM1 also utilizes the acetylation of lysine residues at the N-terminus of APE1 to alter its binding, thereby regulating the nucleolar/nucleoplasmic localization of APE1 and affecting the spatiotemporal control of BER (López et al., 2020a; Scott and Oeffinger, 2016). NPM1 enhances APE1’s cleavage activity at AP sites and also increases the activities of other key BER proteins (Sekhar and Freeman, 2023). Conversely, loss of NPM1 impairs the activities of multiple enzymes within the BER pathway (Poletto et al., 2014). In the TLS pathway, DNA polymerase η is also released into the nucleoplasm, while NPM1 binds to its catalytic core domain, preventing its degradation and thereby facilitating its recruitment to stalled replication forks to perform translesion synthesis (Taha and Ahmadian, 2024; Ziv et al., 2014).

NPM1 also functions as a scaffold protein in multiple steps of DNA repair. In double-strand break (DSB) repair, the E3 ubiquitin ligases RNF8 and RNF168 decorate histones surrounding the damage sites with extensive K63 polyubiquitin chains. Although DSB inducers such as ionizing radiation do not trigger the overall translocation of NPM1, a subset of NPM1 phosphorylated at Thr199 by CDK2/Cyclin E is still able to be recruited to the damage sites via its unique A3 acidic region and ubiquitin-like interacting motif. This process is crucial for both the completion of DNA repair and the disassembly of repair complexes (Koike et al., 2010; Simonetti et al., 2021; Sekhar et al., 2011). Daniel and colleagues have suggested that NPM1 may act as an H3/H4 histone chaperone, responsible for correctly relocating histones after DNA repair to restore normal chromatin structure. This enables local nucleosome reassembly, facilitates the progression of homologous recombination or non-homologous end joining, and ultimately reestablishes higher-order DNA architecture (Scott and Oeffinger, 2016; Colombo et al., 2005; Koike et al., 2010). NPM1, when SUMOylated at the K263 site, can directly interact with RAP80 within the BRCA1-A complex. This interaction regulates the recruitment of the BRCA1-A complex to damage sites. Simultaneously, NPM1 also binds directly to BRCA2 and thereby promotes the formation of repair foci by the key repair protein RAD51 at the site of damage (Sekhar and Freeman, 2023).

Additionally, NPM1 systematically upregulates the expression of DNA damage repair genes by modulating transcription factor activity. A key pathway involved in this regulation is the PP1β-NPM1-retinoblastoma protein (pRB)-E2F1 axis (Lin et al., 2010). UV damage activates the protein phosphatase PP1β and induces its interaction with NPM1, leading to the dephosphorylation of NPM1 at Thr199 and Thr234/237 (Lin et al., 2010). Dephosphorylated NPM1 shows enhanced binding affinity for the pRB. This interaction displaces pRB from the E2F1 transcription factor, which it normally suppresses (Lin et al., 2010). Released E2F1 is then activated and transcriptionally induces the expression of DNA repair-related genes (Lin et al., 2010). In addition, the overexpression of NPM1 can also upregulate the promoter activity and protein level of PCNA, which is an indispensable cofactor in DNA replication and various repair pathways, including nucleotide excision repair (NER) and TLS (Wu et al., 2002). Through this mechanism, NPM1 translates DNA damage signals into the programmed upregulation of repair genes, thereby augmenting the cell’s overall repair capacity at the source.

The role of NPM1 role in DNA repair is not static; its function varies depending on the cell type, signaling pathways, and its own state. For example, in the cytoplasm of endothelial cells, NPM1 forms a ternary complex with p38 and PP2A. During oxidative stress, PP2A is activated and dephosphorylates NPM1 at the Thr199 site. This causes NPM1 to be released from the complex and rapidly translocate into the nucleus, where it suppresses DNA repair (Guillonneau et al., 2016). This observation suggests that NPM1 can exert divergent, even opposing, regulatory functions across distinct cellular lineages.

In AML, the common NPM1c + mutation leads to aberrant cytoplasmic localization of NPM1, which prevents it from effectively stabilizing DNA polymerase η and BER proteins in the nucleus (Ziv et al., 2014). Consequently, key repair proteins are subjected to proteolytic degradation or mislocalization, leading to impaired DNA repair capacity (Ziv et al., 2014; Sekhar and Freeman, 2023). Rather than delving into the specific chemotherapeutic vulnerabilities of leukemia, this mutant paradigm provides a crucial conceptual insight: the proper nucleocytoplasmic distribution of NPM1 is an absolute prerequisite for genomic stability. As observed in various chronic inflammatory diseases, stress-induced spatial dysregulation of NPM1—even without genetic mutations—may similarly disrupt DNA repair complexes, thereby serving as a common pathological link in driving cellular senescence or apoptosis during chronic tissue remodeling.

### 2.3 Regulation of epigenetic modifications

As one of the most abundant nuclear proteins, NPM1’s functions extend far beyond early perceptions of its role in ribosome assembly (Lindström, 2011). For instance, within the intricate network of DDR, NPM1 not only acts as a direct participant in DDR but also serves as a critical signal transducer. It accurately translates genotoxic stress signals, such as DNA damage, into chromatin-level structural remodeling and alterations in gene expression programs. As the carrier of genetic information, the repair, transcription, and other epigenetic dynamics of chromatin serve as the regulatory foundation for the precise execution of all DNA-related processes (Eitoku et al., 2008). Therefore, gaining a deeper understanding of how NPM1 regulates the epigenetic landscape represents a critical bridge connecting its specific molecular physiological functions to its macroscopic effects at the cellular biology level.

As a multifunctional node within the epigenetic regulatory network, NPM1 often exerts its physiological functions through dynamic interactions with histones and key epigenetic modifying enzymes or complexes, leveraging its intrinsic roles as a histone chaperone and reader. Its functional state is tightly coupled to its oligomeric form. NPM1 commonly exists as stable oligomers in the nucleolus, where it induces ribosome biogenesis (Kim et al., 2015). However, its monomeric form preferentially interacts with chromatin regulators such as DOT1L in the nucleoplasm. This spatial distribution and functional differentiation reflect the precision of its regulatory mechanisms. Notably, NPM1 binds to DOT1L and suppresses DOT1L activity, while DOT1L promotes NPM1 transcription. After knocking down NPM1, DOT1L expression is upregulated and exerts its histone methyltransferase activity by increasing H3K79me2 modifications on the *Ezh2* gene. This leads to a substantial upregulation of Ezh2, an enzyme that establishes the H3K27me3 mark. As a result, the inhibitory H3K27me3 mark increases globally, causing the silencing of DNA repetitive elements in perinucleolar heterochromatin (PNH) and ultimately disrupting heterochromatin. This disruption manifests as satellite DNA foci merging into fewer, larger, and disordered clusters, accompanied by nucleolar fragmentation (Izzo et al., 2023).

As an efficient histone chaperone, NPM1 plays a foundational role in shaping and maintaining the dynamic equilibrium of chromatin structure and function (Lindström, 2011; Eitoku et al., 2008). It directly binds to core histone H3-H4 and H2A-H2B heterodimers, facilitating nuclear transport and storage of histones to prevent from aberrant aggregation or degradation in the nucleus, thus ensuring histone supply and chromatin integrity (Eitoku et al., 2008). Recent studies have shown that NPM1 can also bind to histone variants such as HP1BP3, regulate their nucleosome-binding activity, and differentially guide them to genomic regions with distinct epigenetic states. This reveals its role as an active regulator in maintaining higher-order chromatin architecture (Hisaoka et al., 2025).

The NPM1 protein contains one or multiple acidic domains rich in aspartic acid and glutamic acid residues. This structural feature enables it to preferentially bind to the H3K4me2 modification on histones, with the binding affinity increasing as the length of the acidic sequence extends (Wu et al., 2017). Therefore, NPM1 is able to translate the epigenetic information encoded by H3K4me2 into precise chromatin localization and transcriptional activation effects. This specific recognition capability provides a precise molecular basis for NPM1 to target and regulate specific genomic regions such as rDNA (Wu et al., 2017). Through these various interactions, NPM1 participates in multiple critical processes, including nucleosome assembly and disassembly, the formation of higher-order chromatin architecture, and the proper storage and transport of histones within the nucleus (Eitoku et al., 2008; Okuwaki et al., 2012).

As a histone chaperone, NPM1’s function is closely tied to its structural conformation. NPM1 can independently form pentameric structures, which are essential for its robust histone chaperone functions, enabling it to effectively mediate nucleosome assembly and chromatin remodeling (Okuwaki et al., 2012).

NPM1 can directly interact with histone acetyltransferases (HATs) to regulate histone acetylation levels, exhibiting a dual nature in this process. For example, NPM1 directly interacts with GCN5 to inhibit its HAT activity while also competing with GCN5 for histone binding to suppress the histone’s function. During cell mitosis, cyclin-dependent kinases such as CDC2/CDK phosphorylate NPM1 at Thr199, thereby enhancing its inhibitory effect on GCN5. This ultimately maintains histones in a deacetylated and tightly packed state (Zou et al., 2008). Meanwhile, NPM1 can also act as a cofactor for HATs, enhancing their acetylation activity and thereby promoting transcriptional activation of genes (Eitoku et al., 2008). For instance, in oral squamous carcinoma, nitric oxide induces the overexpression of NPM1 and GAPDH. Both NPM1 and GAPDH enhance the HAT activity of p300 by binding to it, thereby activating gene transcription (Arif et al., 2010). This bidirectional regulatory capacity highlights the complexity of NPM1’s fine-tuned and differential regulation across various cellular signaling pathways and gene loci.

Beyond regulating individual nucleosomes, NPM1 organizes and coordinates chromatin dynamics at a higher order through interactions with large multiprotein complexes. Proteomic studies have systematically revealed that NPM1 can physically and functionally interact with multiple key ATP-dependent chromatin remodeling complexes, including the NuRD, P/BAF, and ISWI family complexes (Darracq et al., 2019). In these interactions, NPM1 likely plays a chaperone role, responsible for recruiting or stabilizing these chromatin remodeling complexes to specific genomic loci. This allows for the dynamic regulation of chromatin accessibility, switching between open and closed states, in response to different cellular signals and for precise regulation of gene expression (Darracq et al., 2019).

NPM1 also plays a critical role in maintaining higher-order genomic architecture, particularly the spatial conformation of PNH (Holmberg Olausson et al., 2014). Loss of NPM1 results in pronounced rearrangement and disassembly of PNH (Holmberg Olausson et al., 2014). Mechanistically, NPM1 interacts with heterochromatin the key HP1γ to anchor heterochromatic regions rich in repressive histone marks around the nucleolar periphery (Holmberg Olausson et al., 2014). This structural maintenance function is essential for safeguarding overall genomic stability, preserving higher-order chromatin architecture, and ensuring the organized arrangement of the nucleus.

Under pathological conditions, the normal epigenetic functions of NPM1 are frequently hijacked. The most striking example is found in hematological malignancies, where mutations or chromosomal translocations in NPM1 systematically reprogram the cellular epigenetic landscape. In AML, the cytoplasmic NPM1c + mutant disrupts the nuclear availability of the transcription factor PU.1, switching its co-factors from activators to repressors like DNMT1, thereby systemically blocking myeloid differentiation (Gu et al., 2018). Furthermore, NPM1c + profoundly reshapes the transcriptome by enhancing the Menin-KMT2A complex to hypermethylate HOX genes (Bi and Zhou, 2025), while simultaneously disrupting non-coding RNA networks (such as upregulating lncRNAs like HOXBLINC (Zhu et al., 2021) and downregulating tumor-suppressive miRNAs like miR-204 (Garzon et al., 2008)). It also stabilizes the m6A demethylase FTO, further amplifying oncogenic signaling (Xiao et al., 2022).

**FIGURE 2:**
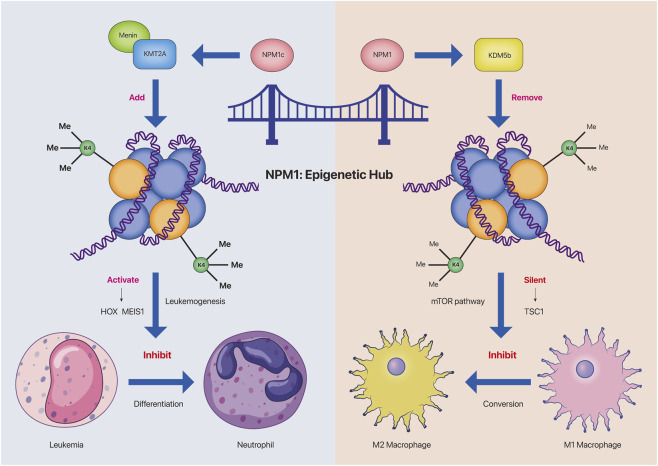
Context-dependent pleiotropy of NPM1 in epigenetic remodeling across distinct disease states. In AML (left), the mutant NPM1c^+^ recruits the Menin-KMT2A complex, leading to increased H3K4me3 marks on HOX gene loci and subsequent blockade of hematopoietic differentiation. Conversely, in the context of myocardial infarction (right), wild-type NPM1 recruits the histone demethylase KDM5b to remove H3K4me3 marks from the TSC1 gene, thereby activating the mTOR pathway. These distinct pathways illustrate NPM1’s versatile role as a central epigenetic hub capable of driving diverse pathological outcomes via H3K4me3 modulation.

The core function of NPM1 as an epigenetic scaffold to regulate differentiation, however, is not confined to these malignant mutations; its interacting networks are highly context-dependent (Figure 2). During the post-myocardial infarction repair process, macrophage phenotype reprogramming plays a crucial role. An important study found that wild-type NPM1 regulates macrophage metabolism and function through epigenetic mechanisms: NPM1 recruits the histone demethylase KDM5b, leading to the removal of H3K4me3 marks from the promoter region of its target gene TSC1, thereby silencing it (Zhang et al., 2024). This in turn relieves TSC1-mediated repression of the mTOR pathway, ultimately sustaining the pro-inflammatory metabolic phenotype M1 in macrophages and preventing their transition to the reparative phenotype M2. Rather than indicating a shared molecular pathway, this contrast elegantly illustrates the profound pleiotropy of NPM1: in AML, mutant NPM1c + recruits the Menin-KMT2A complex to increase H3K4me3 marks on HOX genes; whereas in myocardial infarction, wild-type NPM1 recruits KDM5b to remove H3K4me3 marks on the TSC1 gene. Although the genetic contexts (mutant vs. wild-type) and the recruited epigenetic modifiers are entirely distinct, both scenarios demonstrate how NPM1’s expansive interactome can dictate cell fate via H3K4me3 remodeling, driving the progression of chronic diseases.

Collectively, NPM1 converts upstream physiological and pathological signals—whether they be DNA damage, nutritional stress, or oncogenic mutations—into systemic alterations in chromatin architecture, histone modifications, and RNA regulatory networks. These intricate epigenetic settings clarify the core mechanisms of NPM1 under stress, providing a solid molecular foundation for understanding its downstream functional phenotypes—such as autophagy, apoptosis, and differentiation—in the pathological progression of NCDs discussed in the following section.

## 3 Functional phenotypes of NPM1 in the pathophysiology of chronic diseases

The diverse functional phenotypes and underlying molecular mechanisms of NPM1 across various chronic disease models are summarized in Table 1.

**TABLE 1:** Summary of NPM1-mediated cellular functions and underlying mechanisms across diverse disease models.

Biological function	Disease/Cell model	Key molecular mechanisms	Biological outcome	References
DNA Damage Repair (DDR)	Oxidative stress/Endothelial aging	Disassembly of p38/NPM/PP2A complex & binding to HO-1	Cytoprotective: nuclear retention, preservation of DNA repair, and senescence delay	Guillonneau et al. (2016), Luo et al. (2021)
	Acute myeloid leukemia (AML, NPM1c^+^)	Cytoplasmic mislocalization of NPM1c^+^ → degradation of Pol η and APE1	Impaired DDR: genomic instability and altered sensitivity to chemotherapy	Ziv et al. (2014), Sekhar and Freeman (2023)
Epigenetic regulation	Post-myocardial infarction	NPM1 → recruitment of histone demethylase KDM5b → removal of H3K4me3 on TSC1	Silencing of TSC1, mTOR activation, and macrophage M1 polarization	Zhang et al. (2024)
	AML, NPM1c^+^	NPM1c^+^ → recruitment of Menin-KMT2A/interaction with lncRNAs (e.g., HOXBLINC)	Global chromatin remodeling, increased H3K4me3 at HOX promoters, and leukemogenesis	Bi and Zhou (2025), Zhu et al. (2021), Brunetti et al. (2018)
Protein quality control & LLPS	Amyotrophic lateral sclerosis (ALS)	C9orf72 poly-PR infiltration → disruption of NPM1 liquid-liquid phase separation (LLPS)	Loss of nucleolar protein quality control and systemic dispersal of NPM1	White et al. (2019), Chen et al. (2021), Van Nerom et al. (2024)
	Vascular malformations/Endothelial cells	DDX24 mutation → disruption of NPM1 phase behavior	Collapse of nucleolar homeostasis and aberrantly enhanced endothelial migration	Zhang et al. (2023)
Metabolic reprogramming	NAFLD/Hepatic insulin resistance	Palmitic acid exposure → NPM1 upregulation → NF-κB pathway hijacking	Aggravation of insulin resistance, glucose uptake impairment, and lipid accumulation	Wang X. et al. (2019)
	Metabolic syndrome/Hyperglycemia	Activation of IGF2/IR/NPM1 signaling axis	Immunometabolic reprogramming: shifting macrophages to a pro-inflammatory glycolytic state	Jang et al. (2024)
	Ischemia-reperfusion/Graft kidney	Lactate accumulation → AARS1-catalyzed lactylation at NPM1 K257	Epigenetic rewiring that triggers synchronized ferroptotic waves and tissue loss	Yu et al. (2025)
Tissue remodeling	Pulmonary hypertension/Vascular remodeling	Granzyme B-mediated specific cleavage of NPM1 in differentiated VSMCs	Drives irreversible intimal hyperplasia and vascular wall stiffening	Ulanet et al. (2004), Ulanet et al. (2003)
Autophagy	AML, NPM1c^+^	NPM1c^+^ → stabilization of FTO → upregulation of TP53INP2	Enhancing autophagy and promoting leukemic cell survival	Huang et al. (2023)
		NPM1c^+^ → TRAF6 → ubiquitin-dependent stabilization of ULK1	Enhancing autophagy initiation and promoting cell survival	Tang et al. (2021)
		NPM1c^+^ → binding to GABARAP → activation of transcription factor TFEB	Promotion of lysosomal biogenesis and autophagy	Mende et al. (2023)
	Neurodegenerative disease	Nucleolar stress → NPM1 translocation → p53/PTEN axis → inhibition of mTOR	Induction of neuroprotective autophagy and delayed neuronal death	Kreiner et al. (2013)
	Cataract	miR-429-mediated suppression of NPM1 → modulation of TGF-β2 signaling	Regulation of autophagy and EMT in lens epithelial cells	Bao et al. (2024)
Cell death	Ischemic injury (kidney/heart)	Ischemia → phosphorylation-driven NPM1 translocation to the cytoplasm → binding of Bax	Pro-apoptotic: mitochondrial damage and cytochrome c release	Wang et al. (2013), Kerr et al. (2007), Wang Z. et al. (2019)
​	Hepatocellular carcinoma/Breast cancer	Nuclear NPM1 → sequestration of p53/p14ARF	Anti-apoptotic: preventing mitochondrial translocation or degradation of p53	Dhar and St Clair (2009), Lo et al. (2013)
	Anaplastic large cell lymphoma (ALCL)	NPM-ALK → PI3K/Akt pathway → phosphorylation of Bad/upregulation of Bcl-xL	Anti-apoptotic: preservation of mitochondrial integrity	Greenland et al. (2001), Coluccia et al. (2004)
	Rhabdomyosarcoma/Ewing sarcoma	AURKA/B → phosphorylation of NPM1 → stabilization of SP1/YAP1 → suppression of ACSL5	Anti-ferroptotic: inhibition of lipid peroxidation	Chen et al. (2025), Chen et al. (2024)
	Chronic kidney disease (CKD)	Upregulation of NPM1 → activation of the Nrf2-dependent antioxidant pathway	Anti-ferroptotic: reducing ROS and exacerbation of renal fibrosis	Fu et al. (2025)
	AML, NPM1c^+^	Cytoplasmic NPM1c^+^ → sequestration of Caspase-2	Inhibition of apoptosis: shift of Caspase-2 toward pro-proliferative functions	Sakthivel et al. (2024)
Cell proliferation	Solid tumors	NPM1–Myc positive feedback loop/PI3K–Akt–mTOR pathway	Enhancing ribosome biogenesis and cell cycle progression	Yu et al. (2021), (Li et al. (2008)
	Hepatic progenitor cells (HPCs)	NPM1 → maintenance of mTOR signaling	Promotion of aberrant HPC proliferation	Wang et al. (2024)
​	AML, NPM1c^+^	NPM1c^+^ → upregulation of lncRNAs HOTAIRM1/MALNC	Promotion of leukemic cell proliferation	Jing et al. (2021), Cozzi et al. (2025)
	Diabetic nephropathy	AMPK activation → nucleolar anchoring disruption of NPM1	Suppressing ribosome biogenesis and mesangial cell proliferation	Kodiha et al. (2014)
	AML, NPM1c^+^	NPM1c^+^ → recruitment of Menin-KMT2A → increased H3K4me3 at HOX/MEIS1 promoters	Differentiation arrest: maintenance of hematopoietic stem/progenitor state	Bi and Zhou (2025), Brunetti et al. (2018)
	Myocardial infarction	NPM1 → recruitment of KDM5b → reduced H3K4me3 at the TSC1 promoter → activation of mTOR	Differentiation blockade: macrophage arrest in the pro-inflammatory M1 state	Zhang et al. (2024)

### 3.1 Role of NPM1 in autophagy

As a critical catabolic process for cell survival under various stress conditions, autophagy plays a pivotal role in maintaining cellular homeostasis and function (Gatica et al., 2015). The nucleolus, as a cellular stress sensor, has its functional integrity closely linked to cell survival. Dysregulation of ribosome biogenesis triggers nucleolar stress, which acts as an upstream stimulus for activating autophagy via both p53-dependent and -independent pathways (Pfister, 2019). As a multifunctional nucleolar protein and molecular chaperone, NPM1 plays a complex and pivotal role in cellular stress responses and autophagy regulation. Studies indicate that during nucleolar stress induced by the inhibition of RNA polymerase I transcription, the involvement of the nucleolar protein NPM1 is essential for the initiation of autophagy (Katagiri et al., 2015). This, however, differs from classical starvation-induced autophagy, which does not depend on NPM1.

In NPM1-mutant AML, NPM1 mutations cause the mislocalization of the NPM1c + protein to the cytoplasm, representing one of the most common genetic alterations in this context. These leukemic cells are highly dependent on autophagic activity to sustain their survival. The fat mass and obesity-associated protein FTO is an m6A RNA demethylase. Recent studies have shown that under cardiovascular stress such as hyperglycemia, overexpression of FTO leads to demethylation of *ULK1* mRNA and suppresses *ULK1* mRNA degradation in an m6A-YTHDF2-dependent manner, thereby activating autophagy and attenuating endothelial injury (Xie et al., 2024). Another study has also found that vascular smooth muscle cells sulfhydrate and activate TFEB by secreting cystathionine γ-lyase–derived H2S to enhance autophagic flux. Thus, maintaining high levels of FTO, ULK1, and TFEB is crucial for autophagy (Chen et al., 2022).

NPM1 enhances autophagy through multiple synergistic mechanisms, and it can directly regulate the aforementioned core autophagy proteins. NPM1c + can directly interact with ULK1 and promote TRAF6-dependent K63-linked ubiquitination to stabilize ULK1 protein and sustains its kinase activity (Tang et al., 2021). Furthermore, NPM1c + also interacts with the GABARAP subfamily proteins through an atypical binding module at its N-terminus, thereby activating the transcription factor TFEB to promote autophagy and lysosome biogenesis (Mende et al., 2023).

NPM1 can also indirectly regulate pro-autophagy pathways. NPM1c + interacts with FTO, synergistically upregulating the expression of the autophagy receptor TP53INP2 and promoting its cytoplasmic accumulation to enhance autophagic flux (Huang et al., 2023). Additionally, NPM1c + can bind to the tumor suppressor PML in the cytoplasm, leading to aberrant PML accumulation and activation of autophagy through the AKT signaling pathway (Zou et al., 2017). NPM1c + can bind to the E3 ubiquitin ligase MID1, preventing its degradation of RASGRP3. The stabilized RASGRP3 activates the EGFR-STAT3 signaling cascade to promote both autophagy and proliferation (Wang et al., 2023). Furthermore, NPM1c + stabilizes the transcription factor KLF5 to upregulate the lncRNA HOTAIRM1. In the nucleus, HOTAIRM1 facilitates EGR1 degradation, while in the cytoplasm, it upregulates ULK3 expression by acting as a sponge for miR-152–3p, collectively promoting autophagy and cell proliferation (Jing et al., 2021).

The association between NPM1 and autophagy is not confined to AML. In neurodegenerative diseases such as Huntington’s disease, nucleolar stress, marked by NPM1 dysregulation, triggers a p53/PTEN-dependent neuroprotective autophagy response during the early stages of the disease. This response delays neuronal death by inhibiting the mTOR signaling pathway (Kreiner et al., 2013). In cataracts, the miR-429/NPM1 axis is similarly involved in regulating autophagy and epithelial-mesenchymal transition (EMT) in lens epithelial cells induced by TGF-β2 (Bao et al., 2024). Recent studies have shown that under stress, NPM1 in cardiac progenitor cells is released extracellularly via an autophagy-based unconventional secretion mechanism. This finding not only indicates an early and critical stress response but also provides valuable mechanistic insight for NPM1’s direct involvement in cardiovascular autophagy (Beji et al., 2021).

In summary, NPM1 serves as a central hub protein linking nucleolar stress to the autophagy regulatory network, and its pro-survival function largely depends on the fine-tuned modulation of autophagy. NPM1 can doubly stabilize and activate autophagy at both the post-transcriptional (Huang et al., 2023) and post-translational (Tang et al., 2021) levels. Simultaneously, it also comprehensively enhances the autophagy-lysosome pathway by regulating key transcription factors such as TFEB. Given that ULK1, FTO, and TFEB are core autophagy regulators essential for cellular metabolism and homeostasis, and their dysregulation is closely implicated in the development of multiple chronic inflammatory diseases (Kreiner et al., 2013; Xie et al., 2024; Chen et al., 2022), a highly promising hypothesis emerges. NPM1 may function as a master switch, translating upstream nucleolar stress signals into the regulation of key autophagy proteins like ULK1, FTO, and TFEB, thereby reshaping cellular metabolism and survival strategies. Elucidating the similarities and differences in the role of the NPM1-autophagy regulatory axis across distinct NCDs and verifying its potential as a common therapeutic target represents a highly valuable direction for future research.

### 3.2 The role of NPM1 in cell death

Cell death is one of the core mechanisms maintaining multicellular homeostasis, and its regulatory networks must operate with precision and accuracy (Candoni and Coppola, 2024). As a key node within these networks, NPM1 plays a dual role in different signaling pathways and diseases (Box et al., 2016). Therefore, gaining a deeper understanding of the contextual decision-making mechanisms of NPM1 in cell death not only reveals the profound complexity of biological processes but also opens new avenues for treating cancer, chronic metabolic diseases, and other proliferative disorders.

#### 3.2.1 Regulation of apoptosis by NPM1

Under diverse cellular stress conditions, such as ischemic injury, NPM1, which is primarily located in the nucleolus, rapidly translocates to the cytoplasm (Avitabile et al., 2011; Beji et al., 2021; Wang et al., 2013). This represents a very early apoptotic event, regulated by Bax and Bak through a novel upstream mechanism that is not inhibited by the anti-apoptotic protein Bcl-xL (Lindenboim et al., 2010). In the cytoplasm, NPM1 functions as a Bax chaperone, specifically recognizing and binding to Bax whose spatial conformation has been activated (Kerr et al., 2007). The molecular switch underlying this functional conversion has been elucidated in renal ischemia models, where ischemic stress confers NPM1 a unique phosphorylation fingerprint, a highly consistent and specific alteration in its phosphorylation pattern at five specific serine/threonine sites (T86, S88, T95, T234, and S242), thereby driving NPM1 translocation from the nucleus, depolymerization into monomers, and ultimately its binding to Bax (Wang Z. et al., 2019). Subsequently, as a cofactor for Bax, NPM1 efficiently targets and accumulates on mitochondria after forming a complex with Bax, markedly enhancing Bax’s pro-apoptotic activity (Zhou et al., 2024). This ultimately leads to cytochrome c release and activation of the Caspase cascade (Wang et al., 2013; Kerr et al., 2007). In cases of DNA damage, cytochrome c can also translocate into the nucleus and directly bind to NPM1 in the nucleolar, triggering a conformational change in NPM1. This change leads to the release of ARF protein, which further activates the p53 pathway and promotes apoptosis. This finding reveals a critical role for NPM1 in the cytochrome c-induced, non-mitochondrial-dependent apoptotic process (Geng et al., 2010; Zhou et al., 2024). Another study has shown that after staurosporine treatment in neural precursor cells, NPM1 acts as a scaffold on the mitochondrial surface to facilitate the interaction between p53 and activated Bax, forming a pro-apoptotic complex that triggers mitochondrial apoptotic pathway independent of p53 transcription (Geng et al., 2010).

Conversely, in various tumor cells such as hepatocellular carcinoma, breast cancer, and certain lymphomas, as well as in the pathological contexts of some chronic inflammatory diseases, NPM1 plays a crucial role as a survival guardian, suppressing mitochondrial apoptotic pathway through multiple mechanisms. For instance, nuclear NPM1 can bind to p53 and prevent its translocation to mitochondria, thereby inhibiting p53 from executing its transcription-independent pro-apoptotic functions, even as p53 transcriptionally upregulates Bax (Dhar and St Clair, 2009). NPM1 can also directly bind to the N-terminal region of p53 to inhibit its phosphorylation at the Ser15 site, while simultaneously interacting with p14 ARF to indirectly promote HDM2-mediated ubiquitination and degradation of p53 (Qi et al., 2008). In hepatocellular carcinoma cells, stress-induced NPM1 translocation from the nucleolus to the cytoplasm enables NPM1 to physically bind to Bax, preventing its mitochondrial translocation and activation. This anti-apoptotic effect is independent of the p53-mediated apoptotic pathways (Lo et al., 2013).

In lymphomas driven by the NPM-ALK fusion oncoprotein, the anti-apoptotic pathways of NPM-ALK exhibit notable specificity. These pathways are not only ineffective against exogenous Fas signaling but also unrelated to PLC-γ-dependent pro-proliferative pathways (Greenland et al., 2001), while showing heterogeneity in their relationship with the PI3K/Akt pathway (Greenland et al., 2001; Coluccia et al., 2004; Park et al., 2005). NPM-ALK can upregulate the anti-apoptotic protein Bcl-xL and activate PI3K/Akt signaling, leading to the phosphorylation and inactivation of the pro-apoptotic protein Bad, thereby preventing its binding to Bcl-xL (Coluccia et al., 2004). Simultaneously, NPM-ALK upregulates the heat shock protein Hsp72 to suppress Caspase-3 activity and inhibits the activation and mitochondrial translocation of Bax (Bonvini et al., 2012). Additionally, it activates signaling pathways such as MAPK/ERK and STAT5 to elevate the expression of BCL-2 family proteins, thereby preserving mitochondrial membrane integrity (Greenland et al., 2001). Through these mechanisms, NPM-ALK indirectly protects mitochondria and ultimately suppresses apoptosis while conferring resistance to chemotherapeutic agents.

#### 3.2.2 Regulation of other cell death pathways by NPM1

NPM1’s regulation of cell death extends far beyond mitochondrial apoptosis. In recent years, it has been found to be widely involved in other forms of cell death, demonstrating its complexity as a regulatory hub. Studies have shown that using N6L, a ligand targeting NPM1 on the surface of tumor cells, can directly induce Caspase-3/7-dependent cell death (Destouches et al., 2011). Another study found that cadmium can bind the M/D-rich region of NPM1 to induce nucleolar stress, causing NPM1 to translocate from the nucleolus to the nucleoplasm and reducing pre-ribosomal RNA levels, ultimately triggering cell death (Yan et al., 2025).

Post-translational modification of NPM1 modulates its role in the resistance or sensitivity to distinct cellular death pathways in a tumor type-specific manner. In rhabdomyosarcoma, thereby repressing ACSL5 transcription to resist apoptosis and ferroptosis (Chen et al., 2025). In Ewing sarcoma, AURKB phosphorylates NPM1 at Thr95, enhancing NPM1-YAP1 interaction and stabilizing YAP1 to confer resistance against apoptosis and ferroptosis (Chen et al., 2024). In chronic kidney diseases, NPM1 activates the Nrf2 pathway to suppress apoptosis and ferroptosis, thereby exacerbating renal fibrosis (Fu et al., 2025).

In NPM1-mutant AML, NPM1c + can hijack the pro-apoptotic protein Caspase-2, relocating it to the cytoplasm. This leads to the loss of its pro-apoptotic function and instead supports the proliferation and self-renewal of leukemia cells, revealing a novel mechanism for apoptosis evasion (Sakthivel et al., 2024). Recent studies have shown that βTrCP1 binds to NPM1, preventing its cytoplasmic entry into the cytoplasm and its ability to protect the apoptotic protein p21, leading to p21 degradation. This allows damaged cells to re-enter the cell cycle, resulting in the accumulation of DNA damage and ultimately leading to cell death (Belmonte-Fernández et al., 2025).

**FIGURE 3:**
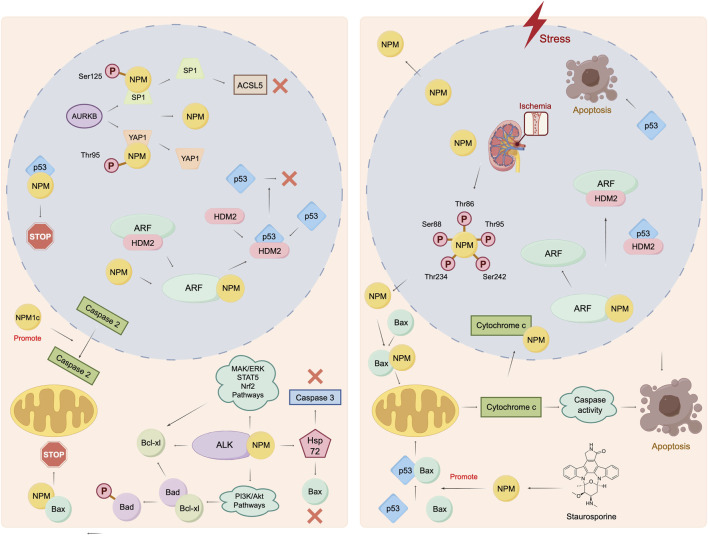
Dual roles of NPM1 in regulating cellular apoptosis under different contexts. Under pro-survival conditions, NPM1 blocks the initiation of the mitochondrial apoptosis program at multiple levels by sequestering p53/p14ARF in the nucleolus, intercepting Bax in the cytoplasm, and activating the PI3K/Akt pathway. In contrast, under adverse stress such as ischemia, NPM1 undergoes specific post-translational modifications that tightly regulate its functional state. For instance, specific phosphorylation events (e.g., at T86, S88, T95, T234, and S242) dynamically modulate its oligomeric state, kinase interactions, and susceptibility to targeted cleavage. Consequently, the modified NPM1 translocates to the cytoplasm, acting as a molecular chaperone for Bax. In this context, NPM1 promotes the accumulation and oligomerization of Bax on the mitochondrial membrane, leading to cytochrome c release and thereby actively driving apoptosis.

In neurons, NPM1 serves as an important survival factor. During nucleolar stress, NPM1 is downregulated while p53 is upregulated, ultimately leading to apoptosis. This pathway is distinct from the canonical p53 signaling cascade, as NPM1 directly activates the mitochondrial apoptosis pathway (Marquez-Lona et al., 2012). NPM1 also frequently exhibits key anti-apoptotic effects in the pathological processes of various non-tumor chronic inflammatory diseases. For instance, in cell injury models stimulated by Dox, such as cardiac progenitor cells (Avitabile et al., 2011), NPM1 overexpression markedly reduced Caspase-8 activity. Moreover, even without Dox stimulation, silencing NPM1 alone leads to apoptosis, indicating the significant anti-apoptotic and cytoprotective roles of NPM1 under stress conditions (Avitabile et al., 2011). Intriguingly, NPM1 is simultaneously secreted extracellularly during its upregulation. However, whether extracellular NPM1 directly regulates apoptosis remains to be elucidated (Beji et al., 2021) (Figure 3).

#### 3.2.3 The molecular mechanisms by which NPM1 regulates cell death

The anti-apoptotic function of NPM1 has become a key target in the development of anti-tumor therapies. In NPM1c + AML, Menin inhibitors such as revumenib and ziftomenib disrupt the oncogenic Menin-KMT2A-NPM1c + axis, downregulate the expression of HOX/MEIS1 and FLT3, thereby relieving differentiation blockage and effectively inducing apoptosis in tumor cells (Perner et al., 2025; Brunetti et al., 2018). Upstream kinases of NPM1, such as LIMK2, promote cancer cell proliferation by phosphorylating the Thr178 site of the MST4 protein, which in turn phosphorylates the Thr95 site of the NPM1 protein, ultimately leading to centrosome clustering (Tian et al., 2025). Experiments have shown that inhibition of LIMK2 also induces mitotic catastrophe and triggers apoptosis (Tian et al., 2025). Likewise, AURKA suppresses apoptosis in rhabdomyosarcoma and Ewing sarcoma cells through the NPM1/SP1/ACSL5 and NPM1/YAP1 pathways, respectively (Chen et al., 2025; Chen et al., 2024). Inhibition of AURKA can reverse these anti-apoptotic effects (Chen et al., 2025; Chen et al., 2024). In hepatic progenitor cells, targeting NPM1 suppresses proliferation and promotes apoptosis by inhibiting signaling pathways, primarily the mTOR pathway (Wang et al., 2024).

NPM1, as a key protein mediating nucleolar stress signaling pathways, has led to the increasing use of nucleolar stress to induce apoptosis in cancer cells as a therapeutic approach in recent years. Recent studies have demonstrate that in pancreatic cancer cells, honey can reduce the expression of multiple nucleolar proteins, such as NPM1, NCL, and FBL, thereby inducing nucleolar stress. This subsequently arrests the cell cycle at the G2/M phase and ultimately triggers apoptosis (Bangash et al., 2024). In NPM-ALK-driven lymphomas, NPM-ALK exhibits kinase activity exclusively in the cytoplasm. In the nucleus, NPM-ALK forms heterodimers with wild-type NPM1, and excessive NPM-ALK can induce oncogenic stress via the MAPK-ERK1/2 pathway, leading to DNA damage and apoptosis. Accordingly, periodically pausing the administration of tyrosine kinase inhibitors may serve as a potential therapeutic strategy to overcome drug resistance and selectively eliminate resistant cells (Ceccon et al., 2016).

### 3.3 Regulation of cell proliferation by NPM1

The determination of cell fate, particularly between proliferation and apoptosis, is precisely regulated by complex signaling networks (Yu et al., 2021). As a central node within these networks, NPM1 is indispensable for cell survival. Genetic ablation of NPM1 can arrest ribosome biogenesis and rapidly trigger cell death (Loubeau et al., 2014). Conversely, NPM1 also promotes survival by antagonizing canonical p53-mediated apoptosis and ferroptosis (Dhar and St Clair, 2009; Chen et al., 2025), and further plays a core role as an active driver of cell proliferation (Chen et al., 2015; Zeng et al., 2018; Li et al., 2017).

The expression level of NPM1 is closely correlated with cellular proliferative activity. Under physiological conditions, NPM1 expression is significantly higher in highly proliferative cells, such as embryonic stem cells, compared to quiescent cells (Guery et al., 2011), while its proliferative potential is strictly constrained by tumor suppressors like p53. NPM1 overexpression in cells with normal p53 expression can instead lead to growth arrest, whereas in the absence of p53, it markedly promotes cell entry into S-phase, thereby accelerating proliferation and even contributing to malignant transformation (Itahana et al., 2003). Under pathological conditions, NPM1 expression is significantly upregulated in various malignant tumors, including solid tumors such as gliomas, gastric cancer, and breast cancer, as well as hematological malignancies (Yu et al., 2021; Chen et al., 2015; Zeng et al., 2018; Zhou et al., 2013; Li et al., 2023; Zheng et al., 2021; Gu et al., 2008; Carlsen et al., 2021; Piao et al., 2017; Coutinho-Camillo et al., 2010; Wang et al., 2022; Luo et al., 2023; Ahuja et al., 2015; Wanzel et al., 2008; Unger et al., 2015; Peng et al., 2017; Qin et al., 2011). This high expression is typically significantly associated with higher tumor grades, later clinical stages, elevated Ki-67 proliferation indices, and poor patient overall survival and recurrence-free survival (Yu et al., 2021; Chen et al., 2015; Zeng et al., 2018; Zhou et al., 2013; Li et al., 2023; Zheng et al., 2021; Coutinho-Camillo et al., 2010; Wang et al., 2022; Luo et al., 2023). Studies have found that the mutation rate of NPM1 is lower in hypoplastic AML compared to non-hypoplastic AML (Carlsen et al., 2021), indicating that NPM1 is not only a biomarker for cell proliferation but also a key driver of tumor progression.

The widespread high expression of NPM1 in tumors is not coincidental; it is precisely regulated at multiple levels, including transcriptional, post-transcriptional, and post-translational regulation. Among these, post-transcriptional regulation mediated by non-coding RNAs is particularly critical. Within non-coding RNAs, especially lncRNAs, the mechanism of competitively binding to microRNAs alleviates the inhibitory effect of miRNAs on the translation of target gene mRNAs. This represents one of the core mechanisms underlying the upregulated expression of NPM1 in various cancers (Li et al., 2023; Shen et al., 2022). For example, in NPM1c + AML, NPM1c + upregulates the lncRNA HOTAIRM1 to sponge miR-152–3p, thereby promoting the proliferation and autophagy of leukemia cells (Jing et al., 2021). Meanwhile, in estrogen-driven endometrial carcinoma, the lncRNA NNT-AS1 sequesters miR-30c, which suppresses NPM1, thereby attenuating miR-30c′s function and leading to NPM1 upregulation and enhanced proliferation (Shen et al., 2022). These lncRNAs promote tumor cell proliferation by sponging corresponding miRNAs, which in turn upregulates NPM1 protein expression.

Furthermore, NPM1-lncRNA can also mediate transcriptional regulation to control proliferation. For instance, a recent study on NPM1c + AML identified the lncRNA MALNC, which binds to chromatin and regulates the expression of a series of genes, including those in the retinoic acid signaling pathway, thereby inhibiting differentiation and promoting proliferation (Cozzi et al., 2025). In the tumor-suppressive pathway involving the lncRNA LINC01088 in esophageal cancer, LINC01088 upregulates the cytoplasmic expression of NPM1 by binding to it. This disrupts its DNA repair function while simultaneously reducing the expression of mutant p53 and enhancing p53 transcriptional activity. This reveals the context-dependent role of NPM1 in proliferation (Liang et al., 2023). Notably, the lncRNA LETN can bind to the C-terminal domain of NPM1, and the pentamer formation of NPM1 depends on LETN. Knockdown of either LETN or NPM1 suppresses ribosome biogenesis and causes proliferation arrest, indicating that LETN is essential for maintaining both the function of NPM1 and nucleolar structure (Wang et al., 2021).

The role of NPM1 in cell proliferation is not limited to interactions with lncRNAs; it is also regulated by upstream transcription factors. In endometrial carcinoma, the transcription factor KLF2 binds to the NPM1 promoter and represses its transcription, thereby exerting a tumor-suppressive effect (Cheng et al., 2023). The mTOR signaling pathway also binds to the NPM1 promoter to activate its transcription. At the post-transcriptional level, it stabilizes the mRNA of NPM1 to upregulate NPM1, ultimately promoting cell proliferation through NPM1’s maintenance of high Cyclin D1 levels (Boudra et al., 2016). In astrocytomas, Rac1, a downstream component of the mTOR pathway, can upregulate NPM1 expression and promote proliferation via enhancing protein synthesis (Sandsmark et al., 2007). Additionally, drug interventions can also repress NPM1 expression at the transcriptional level. For example, Neurolenin B suppresses ALCL cell proliferation and activates proteins like Caspase 3 to promote apoptosis by inhibiting the expression of NPM-ALK, either transcriptionally or through mRNA stability (Unger et al., 2015).

In addition to the upregulation of expression levels, the stability of the NPM1 protein is also tightly regulated by PTMs and protein interactions, which are critical for maintaining its pro-proliferative function. Phosphorylation is a prototypical modification that governs proliferative process. As the primary upstream kinases of NPM1, casein kinase 2 (CK2) and cyclin-dependent kinases (CDKs) play key roles in cell proliferation (Negi and Olson, 2006; Zhao et al., 2015). During interphase, CK2 phosphorylates NPM1 at Ser125 to increase its mobility within the nucleolus (Negi and Olson, 2006). At the onset of mitosis, CDK1 rapidly phosphorylates the C-terminal sites of NPM1 such as Thr199, markedly reducing its affinity for nucleolar components such as RNA. This enables NPM1 to be released from the nucleolus and dispersed throughout the cell, a prerequisite for nucleolar disassembly and the proper progression of mitosis (Negi and Olson, 2006). In the cytoplasm, NPM1 participates in core mitotic events such as chromosome alignment, spindle morphology, and kinetochore-microtubule attachment. Inhibition of NPM1 impairs normal cell division, induces mitotic catastrophe, and activates p53-dependent checkpoint pathway, ultimately causing mitosis to arrest at the G1 phase (Amin et al., 2008). Furthermore, during the M phase, the peptidyl-prolyl cis-trans isomerase Pin1 recognizes phosphorylated NPM1, particularly when it contains the pSer/Thr-Pro motifs, and alters its local three-dimensional conformation to ensure proper progression of mitosis. This highlights the dual regulation of NPM1 in cell proliferation through strict temporal phosphorylation and conformational isomerization during the cell cycle (Zhao et al., 2015). Moreover, during the final stage of cell division, evidence indicates that Aurora B-mediated phosphorylation of NPM1 at Ser125 is crucial for the successful completion of cytokinesis (Shandilya et al., 2014). The above studies demonstrate that phosphorylation of specific sites on NPM1 at different phases of the cell cycle is key to the precise regulation of its diverse functions.

Phosphorylation modifications of NPM1 generally promote cell proliferation in many tumor diseases. In prostate cancer, phosphorylation of NPM1 at Thr199 and Thr234/237 can also cause its overexpression and may be involved in androgen receptor signaling (Destouches et al., 2016). In osteosarcoma, Aurora B activates subsequent pro-proliferation pathways by phosphorylating NPM1 at Ser125 (Song et al., 2020). Meanwhile, evidence also indicates that Aurora B and Ser125-phosphorylated NPM1 are detected in oral cancer (Shandilya et al., 2014). Pharmacologic interventions can alter the phosphorylation state of NPM1 and thereby affect its function. For example, the CDK4/6 inhibitor palbociclib promotes dephosphorylation of NPM1 at Thr199, and dephosphorylated NPM1 inhibits the proliferation of endometrial cancer cells by upregulating the expression of ERα (Lin et al., 2019). N6L can also treat advanced prostate cancer by inhibiting NPM1 phosphorylation (Destouches et al., 2016).

In addition to phosphorylation, the roles of ubiquitination and SUMOylation in the regulation of cell proliferation by NPM1 should not be underestimated. Evidence suggests that the tumor suppressor ARF can induce polyubiquitination and degradation of NPM1 (Itahana et al., 2003; Chao et al., 2013), and this process is p53-independent and represents an distinct function of ARF (Loubeau et al., 2014). Estrogen stabilizes NPM1 by directly suppressing ubiquitination and inhibiting ARF-NPM1 interaction, thereby promoting cell proliferation in an ERα-dependent manner (Chao et al., 2013). Further studies have found that the lysine residues K263 and K230 of NPM1 can be SUMOylated. Mutation at the K263 site prevents NPM1 from localizing to the nucleolus and centrosome. SUMOylated NPM1 is protected from cleavage by Caspase-3, allowing it to more stably exert its pro-proliferative effects. SUMOylated NPM1 binds extensively to Rb, relieving Rb-mediated repression of E2F1, and ultimately upregulating E2F1 to promote proliferation (Liu et al., 2007). In addition to physiological regulation, drug interventions can also alter the ubiquitination status of NPM1 to affect its pro-proliferative function. For example, in NPM-ALK-driven anaplastic large cell lymphoma, arsenic trioxide promotes the ubiquitination and degradation of the NPM-ALK protein by inducing the production of reactive oxygen species (ROS) (Piao et al., 2017).

NPM1 can also enhance its regulatory function in proliferation through protein interactions, a phenomenon commonly observed in tumor development. In pancreatic cancer, the oncoprotein IMUP directly binds to NPM1 and increases its protein stability (Luo et al., 2023). In esophageal squamous cell carcinoma, FAM84 B binds to the C-terminal region of NPM1, enhancing its nuclear localization and expression levels, thereby driving proliferation (Wang et al., 2022). Additionally, during mitosis in embryonic stem cells, NPM1 can bind to Tpt1 to maintain high proliferation rates (Johansson et al., 2010).

Beyond upstream regulation, NPM1 directly drives proliferation by modulating the core cell-cycle machinery. To accelerate the G1/S transition, NPM1 downregulates cycle inhibitors (e.g., promoting p27Kip1 degradation via Skp2, or repressing CDKN2A) and upregulates promoters like Cyclin D1 and CDC25A, enabling cells to efficiently bypass checkpoints (Chen et al., 2015; Zeng et al., 2018; Wang et al., 2022; Qin et al., 2011; Fernandez-Vidal et al., 2009). During S-phase, NPM1 can epigenetically silence FHL1, permitting unchecked CDC25A activity and persistent CDK2/Cyclin A/E1 activation (Luo et al., 2023). However, NPM1’s regulation is highly context-dependent and biphasic. For instance, the cytoplasmic interaction of NPM1 with Miz1 paradoxically induces G1 arrest (Wanzel et al., 2008). Similarly, in gastric cancer, NPM1 promotes GADD45α nuclear translocation to inhibit the Cdk1/Cyclin-B complex, causing a tumor-suppressive G2/M arrest (Lakshmi et al., 2021). Consequently, targeted depletion or pharmacological inhibition of NPM1 or its fusions (e.g., via TFP) predominantly induces a p53-dependent G0/G1 block (Hsu et al., 2007; Uchihara et al., 2021; Hu et al., 2018).

Beyond direct cell-cycle regulation, NPM1 amplifies proliferative signals by functioning as a central hub that sustains and activates multiple classical pro-survival pathways. First, NPM1 engages in a robust positive feedback loop with the oncogene c-Myc; it acts as both a transcriptional target and a molecular chaperone of c-Myc, synergistically modulating growth-related genes to tightly couple ribosome biogenesis with cell-cycle progression (Coutinho-Camillo et al., 2010; Wanzel et al., 2008; Peng et al., 2017; Li et al., 2008; Yung, 2004). Second, NPM1 is critical for sustaining the PI3K/Akt/mTORC1 axis, driving aberrant proliferation and chemoresistance in both hematological malignancies (e.g., NPM1c + AML and ALCL) and solid tumors (Sakthivel et al., 2024; Yu et al., 2021; Coutinho-Camillo et al., 2010; Sandsmark et al., 2007; Uchihara et al., 2021; Gu et al., 2004). Interestingly, NPM1 also serves as a key downstream effector for energy-sensing pathways like AMPK to restrict proliferation during metabolic stress (Kodiha et al., 2014). Furthermore, wild-type NPM1 and its oncogenic mutants (e.g., NPM-ALK) broadly activate an array of mitogenic cascades—including EGFR/MAPK/ERK (Li et al., 2023; Wu et al., 2002), JNK/c-Jun (Leventaki et al., 2007), JAK2/STAT5 (Ruchatz et al., 2003), and ERK/NF-κB (Song et al., 2020)—thereby comprehensively promoting proliferation, migration, and survival across diverse cancer models.

Beyond its role in tumors, NPM1 also plays a complex part in the pathological processes of other NCDs, particularly in terms of cell proliferation and tissue remodeling. For example, in vascular smooth muscle cells (VSMCs), NPM1 expression is upregulated in response to serum and estrogen (Koike et al., 1996). Considering the effects of estrogen on VSMC proliferation, this suggests that NPM1 may be involved in regulating the proliferation of VSMCs during chronic pathological processes such as vascular injury repair or atherosclerosis (Koike et al., 1996). In a model of HUVEC dysfunction induced by oxidized-LDL, NPM1 expression was downregulated alongside the inhibition of cell proliferation (Kinumi et al., 2005), indicating that the expression level of NPM1 is correlated with the proliferative state of endothelial cells. However, other studies have indicated NPM1 secreted into the extracellular space can instead promote angiogenesis and endothelial cell migration and proliferation via TLR4 activation (Beji et al., 2021).

### 3.4 Regulatory roles of NPM1 in cell differentiation

Unlike its indispensable roles in fundamental life activities such as maintaining genome stability, ribosome biogenesis, autophagy, cell death, and proliferation (Guery et al., 2011; Zhang et al., 2012; Solier et al., 2017; Selheim et al., 2024), NPM1 plays complex and even seemingly contradictory multifaceted roles in cell differentiation (Sportoletti et al., 2013). On one hand, normal embryonic development and tissue differentiation requires precise regulation of NPM1, and its deficiency results in severe developmental defects or even embryonic lethality (Johansson and Simonsson, 2010). On the other hand, in hematological malignancies, mutations or translocations in the *NPM1* gene convert it into a potent oncoprotein, whose core pathogenic mechanism is to actively block normal cellular differentiation (Nichol et al., 2016; Leong et al., 2010). Understanding how NPM1 performs its functions across varying physiological and pathological contexts is therefore critical.

#### 3.4.1 Physiological downregulation of NPM1 permits differentiation

NPM1 serves as a key guardian in maintaining the undifferentiated state of cells. In embryonic stem (ES) cells, NPM1 forms protein complexes with the core pluripotency transcription factors Oct4, Sox2, and Nanog, collectively maintaining cell pluripotency (Johansson and Simonsson, 2010). Downregulation of NPM1 expression prompts ES cells to initiate differentiation programs, particularly toward the mesodermal lineage (Johansson and Simonsson, 2010). The fundamental role of NPM1 in development has been further validated in knockout models, where complete loss of Npm1 leads to mid-gestational embryonic death due to severe neurodevelopmental and primitive hematopoietic defects (Qing et al., 2008). In contrast, overexpression of NPM1 in hematopoietic stem cells promotes excessive proliferation while blocking differentiation into mature myeloid cells (Li et al., 2006).

To successfully initiate terminal differentiation, cells must escape the undifferentiated state by employing multilayered mechanisms to reprogram or downregulate NPM1. At the transcriptional level, differentiation-inducing signals can shut down NPM1 expression at its source (Yung, 2004; Hsu and Yung, 2026). At the post-translational level, such as during TPA-induced megakaryocytic differentiation, NPM1 protein is rapidly degraded via the ubiquitin-proteasome pathway (Chou et al., 2007; Hsu and Yung, 2003). This physiological downregulation is also mirrored in various solid tumors; during the induced differentiation of hepatocellular carcinoma, neuroblastoma, and colon cancer cells, NPM1 expression levels consistently decrease, accompanied by significant changes in its subcellular localization and protein-protein interactions (Li et al., 2010; Xu et al., 2014; Shi et al., 2010; Van Belzen et al., 1995).

#### 3.4.2 NPM1 cleavage in macrophage and tissue remodeling

More intriguingly, during the differentiation of monocytes into macrophages—a process highly relevant to the immunometabolic microenvironment of chronic NCDs—NPM1 is not simply degraded. Instead, it is specifically cleaved into 30 kDa and 20 kDa fragments by Caspases (3, 7, and 8) and Cathepsin B (Guery et al., 2011; Jacquel et al., 2009). These N-terminal NPM1 fragments are essential for maintaining the phenotype of quiescent macrophages by restraining inflammatory activity, and they rapidly disappear upon inflammatory activation (Guery et al., 2011; Jacquel et al., 2009).

Differentiation status can also inversely influence NPM1 function in vascular tissues. During the differentiation of vascular smooth muscle cells (VSMCs)—key players in chronic vascular remodeling—the efficiency of NPM1 cleavage by Granzyme B is increased approximately 38-fold compared to undifferentiated cells (Ulanet et al., 2004). In this specific context, NPM1 acts not as an active driver of chronic remodeling, but rather as a sensitive target and sensor of the cellular differentiation state, suggesting its intricate involvement in fibrotic or matrix proliferative diseases (Ulanet et al., 2004).

#### 3.4.3 Malignant hijacking of NPM1 to block differentiation

In stark contrast to its precisely regulated physiological downregulation, NPM1 is frequently genetically altered in hematological malignancies to actively freeze cells in an undifferentiated state. This occurs through chromosomal translocations generating fusion proteins (e.g., NPM1-RARα in APL (Zhang et al., 2000; Pollock et al., 2014; Rush et al., 2013) and NPM-ALK in ALCL (Pawlicki et al., 2021)) or through mutations generating the cytoplasmic NPM1c + variant in AML (Sportoletti et al., 2013).

In approximately one-third of AML patients, mutations generate a cytoplasmic NPM1c + variant that actively blocks differentiation (Sportoletti et al., 2013). A hallmark of this arrest is the NPM1c + -mediated enhancement of the Menin-KMT2A epigenetic complex in the nucleus, which persistently upregulates stemness-associated genes like HOX clusters and MEIS1 (Nichol et al., 2016), (Brunetti et al., 2018). While NPM1c + also disrupts differentiation via other mechanisms—such as inhibiting Caspase-6/8 (Leong et al., 2010), impairing ARID3C (Kim et al., 2024), and upregulating miR-10b (Zou et al., 2016)—its role as an epigenetic scaffold remains central. Therapeutically, the reliance of AML on NPM1-mediated differentiation arrest provides a strong proof-of-concept for targeted intervention. Agents that disrupt the Menin-KMT2A interaction (e.g., Revumenib) (Salman and Stein, 2024; Hogeling et al., 2024), alter NPM1c + subcellular localization (e.g., Selinexor) (Brunetti et al., 2018), or directly target NPM1 oligomerization (e.g., NSC348884) (Balusu et al., 2011; Brunetti et al., 2018) have shown efficacy in reinitiating differentiation and inducing apoptosis. While these specific agents primarily target the mutant NPM1c + or its unique interactome, their clinical success underscores the broader translational potential of targeting NPM1-associated epigenetic hubs in other chronic diseases where wild-type NPM1 drives pathological remodeling.

Crucially, this mutant paradigm highlights the potent capacity of NPM1 to arrest cell fate, setting a conceptual precedent for how wild-type NPM1 might similarly freeze cellular states (such as macrophage polarization) during chronic inflammatory remodeling in other NCDs. Furthermore, NPM1 exhibits important regulatory roles in chronic cardiovascular diseases, with growing attention on its involvement in tissue remodeling and immunometabolic regulation. Recent studies have elucidated a key mechanism by which wild-type NPM1 impedes macrophage differentiation toward a reparative phenotype: in the context of tissue repair following myocardial infarction, stress-responsive NPM1 recruits the histone demethylase KDM5b to the TSC1 promoter, removing H3K4me3 modifications to suppress TSC1 transcription. This subsequently activates the mTOR pathway and locks macrophages into a glycolytic metabolic state, thereby blocking their differentiation into an M2-like phenotype with tissue-repair functions (Zhang et al., 2024). This highlights how pathological remodeling across chronic inflammatory diseases can be driven by stress-induced alterations in wild-type NPM1’s epigenetic regulatory functions, fundamentally distinct from the mutational drivers seen in hematological malignancies.

### 3.5 Emerging roles of NPM1 in cellular senescence and metabolic reprogramming

Beyond apoptosis and differentiation, NPM1 is increasingly recognized as a critical molecular supervisor of cellular senescence, a permanent state of cell-cycle arrest that drives organismal aging and chronic inflammatory NCDs (Luo et al., 2021). Mechanistically, the initiation of cellular senescence is intimately coupled with stress-induced nucleolar dysfunction and the subsequent spatial redistribution of wild-type NPM1. Under chronic low-grade metabolic or genotoxic stress, the progressive depletion or abnormal nucleocytoplasmic translocation of NPM1 disrupts its canonical scaffolding functions within the nucleolus. In unstressed cells, NPM1 protein safely sequesters the tumor suppressor p14ARF (p19ARF in rodents) within the nucleolar compartment. However, the stress-induced dispersion of NPM1 liberates p14ARF into the nucleoplasm, where it directly binds to and inhibits the E3 ubiquitin ligase MDM2. This disruption effectively halts MDM2-mediated p53 degradation, leading to the robust stabilization and activation of the p53-p21CIP1/WAF1 signaling cascade. The persistent activation of this axis locks downstream cyclin-dependent kinases, thereby reinforcing the senescent growth arrest and initiating the transcription of the pro-inflammatory senescence-associated secretory phenotype (SASP).

This NPM1-dependent senescence framework plays a pivotal role in vascular aging and endothelial dysfunction, which form the pathological basis of atherosclerosis and ischemic heart diseases (Luo et al., 2021). During the progression of endothelial senescence, cells employ counter-regulatory mechanisms to modulate NPM1 localization as a defense against premature aging. A key primary study demonstrated that under oxidative or replicative stress, the upstream protective enzyme heme oxygenase-1 (HO-1) undergoes a targeted nuclear translocation (Luo et al., 2021). Once inside the nucleus, HO-1 physically interacts with the N-terminal domain of wild-type NPM1. This specific binding acts as a molecular anchor that restricts NPM1 within the nuclear compartment, preventing its pathological nuclear export and stabilizing the genomic architecture. Consequently, this HO-1-mediated nuclear retention of NPM1 disrupts the aberrant assembly of the pathological NPM1/p53/MDM2 complex, thereby dampening the chronic p53-p21 cascade and effectively delaying stress-induced endothelial senescence (Luo et al., 2021). Conversely, when this protective anchoring network breaks down under persistent environmental insults, the uncontrolled spatial dysregulation of NPM1 accelerates vascular cell aging, fueling the development of age-related chronic vascular disorders.

### 3.6 NPM1 in neurodegenerative diseases: phase separation and nucleolar stress

The nucleolus has recently been redefined beyond its classical role in ribosome biogenesis as a critical nuclear protein quality control (PQC) compartment that dynamically regulates misfolded nuclear proteins via liquid-liquid phase separation (LLPS) (Rekulpally and Suresh, 2020). Within this non-membrane-bound compartment, NPM1 plays a foundational role in maintaining cellular proteostasis by forming liquid-like protein condensates that aid in protein refolding and prevent toxic protein aggregation under proteotoxic stress (Rekulpally and Suresh, 2020). However, this LLPS-mediated nuclear quality control mechanism is severely perturbed during neurodegeneration, particularly in amyotrophic lateral sclerosis (ALS) associated with the C9orf72 hexanucleotide repeat expansion (White et al., 2019; Rekulpally and Suresh, 2020). The C9orf72 expansion encodes toxic arginine-rich dipeptide repeat proteins (R-DPRs), such as proline-arginine (poly-PR), which directly infiltrate the nucleolus and interact with the acidic domains of NPM1 (White et al., 2019; Chen et al., 2021). This aberrant interaction disrupts the electrostatic equilibrium required for physiological phase separation, causing the dissolution of functional NPM1-rRNA droplets and inducing the systemic dispersal of NPM1 throughout the nucleoplasm (White et al., 2019). Crucially, the precise alternate charge distribution of these pathogenic R-DPRs facilitates the multivalent entrapment of NPM1, subsequently undermining its normal molecular diffusion, transcription, and translation within the nucleolar architecture (Chen et al., 2021). Furthermore, these R-DPR-NPM1 condensates tend to undergo a slow liquid-to-solid phase transition into aggregated, Thioflavin S (ThS)-positive states that can actively recruit other ALS-linked proteins like TDP-43, thereby amplifying localized proteotoxicity (Van Nerom et al., 2024). Beyond proteotoxic aggregation, the loss of nuclear NPM1 functional scaffold in ALS severely exacerbates genomic instability, as evidenced by the robust induction of the DDR and the specific inhibition of homology-directed DNA double-strand break repair pathways (Farg et al., 2017; Andrade et al., 2020).

In addition to the physical disruption of biomolecular phase separation observed in ALS, chronic transcriptional and translational exhaustion of NPM1 characterizes the pathology of Parkinson’s disease (PD) (Garcia-Esparcia et al., 2015). In the substantia nigra of PD patients, the progressive accumulation of abnormal $\alpha$-synuclein oligomers strongly correlates with a significant reduction in NPM1 mRNA expression and a marked loss of NPM1 nucleolar immunostaining in remaining dopaminergic neurons (Garcia-Esparcia et al., 2015). This disease-associated downregulation of NPM1 is further substantiated in experimental neurotoxic models, such as 1-methyl-4-phenylpyridinium (MPP+)-treated human SH-SY5Y neuroblastoma cells, where molecular chaperones including NPM1 undergo pronounced expression and structural alterations in response to protein misfolding stress (Yu et al., 2016). A strikingly similar paradigm of nucleolar chaperone system failure is observed in the frontal cortex of patients with Alzheimer’s disease (AD) (Hernandez-Ortega et al., 2017). Quantitative analyses of post-mortem brain tissues in AD patients and corresponding transgenic mouse models exhibit a profound decrease in NPM1 expression levels, which directly impairs the cell’s baseline capacity to synthesize critical ribosomal components and sustain protein synthesis machinery (Hernandez-Ortega et al., 2017). Moreover, in cortical neurons harboring familial AD-linked presenilin-1 (PS1) mutations, the expression of NPM1 mRNA is intrinsically lower and fails to adapt adequately under environmental stress, rendering these mutant neurons uniquely vulnerable to glutamate excitotoxicity and subsequent apoptosis due to altered calcium and chaperone homeostasis (Maezawa et al., 2002). Collectively, these findings demonstrate that whether through the physical disruption of its liquid-liquid phase separation or via chronic expression exhaustion, the loss of functional NPM1 represents a convergent pathological hub that links nucleolar stress and proteoprotective failure to the progression of major non-communicable neurodegenerative diseases (White et al., 2019; Garcia-Esparcia et al., 2015; Hernandez-Ortega et al., 2017; Rekulpally and Suresh, 2020).

### 3.7 NPM1 in metabolic diseases: insulin resistance and metabolic reprogramming

Beyond its documented involvements in cardiovascular and neurodegenerative pathologies, NPM1 is emerging as a critical molecular rheostat that senses nutrient overload and dictates the pathophysiological progression of metabolic disorders, including type 2 diabetes (T2D) (Tang et al., 2025), obesity, and non-alcoholic fatty liver disease (NAFLD) (Wang X. et al., 2019; Jang et al., 2024). In chronic metabolic stress environments characterized by lipotoxicity, elevated levels of circulating free fatty acids, such as palmitic acid, directly stimulate the transcriptional and translational upregulation of wild-type NPM1 in parenchymal cells (Wang X et al., 2019). This aberrant overabundance of hepatic NPM1 actively hijacks and sustains the canonical NF-κB signaling cascade (Wang X et al., 2019). Consequently, the chronic activation of this axis downregulates insulin receptor substrate signaling, thereby severely aggravating hepatic insulin resistance, impairing basal glucose uptake, and driving ectopic lipid droplet accumulation (Wang X. et al., 2019). Concurrently, the systemic hyperglycemia that defines advanced metabolic syndrome serves as a potent upstream stimulus that activates the paracrine insulin-like growth factor 2 (IGF2)/insulin receptor (IR)/NPM1 signaling axis (Jang et al., 2024). By accelerating glucose influx through the transcriptional upregulations of glucose transporters, this hyperglycemia-induced NPM1 activation locks tissue-resident macrophages into a highly glycolytic, pro-inflammatory phenotype, thereby continuously fueling the low-grade chronic inflammation that exacerbates systemic metabolic dysfunctions (Jang et al., 2024).

Furthermore, the metabolic regulatory network of NPM1 intimately intersects with RNA epigenetic modifications and autophagy. The fat mass and obesity-associated protein (FTO), an m6A RNA demethylase tightly regulated by NPM1 networks, plays a pivotal role under metabolic stress. Under cardiovascular stress such as hyperglycemia, FTO activation leads to the demethylation of ULK1 mRNA and suppresses its degradation, thereby activating autophagy and attenuating endothelial injury (Xie et al., 2024). Synergistically, sustaining high levels of FTO and TFEB via paracrine signaling is crucial for maintaining autophagic flux in vascular smooth muscle cells (Chen et al., 2022), suggesting that NPM1-associated epigenetic hubs are intrinsically linked to metabolic autophagy.

Crucially, the most cutting-edge paradigm shifts indicate that metabolic stress does not merely alter NPM1 abundance, but directly rewires its structural capacities through post-translational modifications driven by metabolic byproducts (Yu et al., 2025). Under conditions of hyperactive glycolysis where cellular lactate accumulation reaches a critical threshold, the lactyltransferase AARS1 directly utilizes this byproduct to catalyze lactylation at the lysine 257 (K257) residue of wild-type NPM1 (Yu et al., 2025). Rather than executing its homeostatic functions, K257-lactylated NPM1 functions as a dominant biochemical switch that unleashes synchronized ferroptotic trigger waves through neighboring tissue layers (Yu et al., 2025). Taken together, whether acting as a responsive downstream scaffold to lipotoxic and hyperglycemic stresses or functioning as a direct substrate for lactylation-mediated epigenetic rewiring, wild-type NPM1 operates as an indispensable hub linking systemic metabolic perturbations to chronic inflammatory cell-fate decisions (Wang X. et al., 2019; Jang et al., 2024; Yu et al., 2025).

### 3.8 Spatiotemporal dynamics of NPM1 in cardiovascular remodeling

#### 3.8.1 Context-dependent molecular switches: protective vs. pro-inflammatory triggers

The dualistic nature of NPM1 in the cardiovascular system is tightly governed by the type and severity of upstream stress stimuli, which ultimately dictate its subcellular localization. Under acute or moderate oxidative stress, such as the initial phase of ischemia, NPM1 primarily exerts a cytoprotective role. Upstream mediators like heme oxygenase-1 (HO-1) undergo nuclear translocation and physically interact with the N-terminus of NPM1, retaining it in the nucleus to execute DNA repair and prevent premature endothelial senescence (Luo et al., 2021). Simultaneously, the targeted disassembly of the p38/NPM1/PP2A complex under oxidative stress preserves nuclear homeostasis (Guillonneau et al., 2016). In stark contrast, under chronic lipotoxicity (e.g., hyperlipidemia) or severe genotoxic stress (e.g., doxorubicin exposure), NPM1 undergoes a pathological switch toward a pro-inflammatory driver. These severe stresses induce the irreversible cytoplasmic accumulation of NPM1, where it directly binds to the p65 subunit of NF-κB, activating profound vascular inflammation (Rao et al., 2021). In extreme conditions of cellular injury, NPM1 is even secreted extracellularly as an alarmin (DAMP), binding to TLR4 receptors on neighboring cells to unleash an autocrine/paracrine inflammatory storm (Beji et al., 2021).

#### 3.8.2 The role of NPM1 in cardiac fibrosis and vascular remodeling

This chronic pro-inflammatory switch of NPM1 is fundamentally linked to the progression of cardiac fibrosis and structural remodeling. Although wild-type NPM1 primarily acts as an epigenetic and metabolic regulator in immune cells, its pathological retention in the ischemic microenvironment exerts profound paracrine effects on resident cardiac fibroblasts. By recruiting KDM5b and activating the mTOR pathway, NPM1 locks macrophages in a pro-inflammatory M1-like state (Zhang et al., 2024). These metabolically reprogrammed macrophages continuously secrete profibrotic cytokines (e.g., TGF-β) that paracrinally drive the activation, proliferation, and transdifferentiation of cardiac fibroblasts, leading to excessive collagen deposition and adverse ventricular fibrosis.

Furthermore, within the vascular compartment, NPM1 exhibits exquisite vulnerability to Granzyme B-mediated cleavage specifically in differentiated vascular smooth muscle cells (VSMCs) (Ulanet et al., 2004; Ulanet et al., 2003). This targeted proteolytic disruption of NPM1 actively drives intimal hyperplasia and vascular wall stiffening. Beyond proteolytic cleavage, the disruption of NPM1’s biophysical properties also acts as a critical driver of pathological vascular remodeling. For instance, in vascular malformations, loss-of-function mutations in DDX24 disrupt the LLPS behavior of NPM1 (Zhang et al., 2023). This phase transition failure leads to the collapse of nucleolar homeostasis, aberrantly enhancing endothelial cell migration and tube formation (Zhang et al., 2023). Together, these proteolytic and biophysical dysregulations of NPM1 provide a direct mechanistic link to the fibrotic vascular remodeling and anomalous angiogenesis observed in conditions like pulmonary hypertension and chronic heart failure.

## 4 Therapeutic strategies and challenges in targeting NPM1

Given its central role as a stress integrator, NPM1 presents a highly attractive but challenging therapeutic target. Current pharmacological strategies primarily originate from oncology, such as small-molecule inhibitors targeting NPM1 oligomerization (e.g., NSC348884) or agents disrupting its epigenetic interactome (e.g., Menin inhibitors like Revumenib). While these agents show remarkable efficacy in NPM1-mutant leukemias, translating them into treatments for non-malignant NCDs poses significant conceptual and technical hurdles.

The foremost challenge is preserving its essential physiological functions. Since wild-type NPM1 is indispensable for basic cellular survival (e.g., ribosome biogenesis and histone chaperoning), indiscriminate systemic inhibition of NPM1 would likely induce severe toxicity, mimicking devastating nucleolar stress. Therefore, future therapeutic strategies in NCDs must pivot from “global inhibition” to “precision targeting.” First, this requires the development of drugs that disrupt only specific pathological interactions (such as blocking the NPM1-KDM5b interaction in heart failure) without affecting its basal chaperone functions. Second, targeting the upstream kinases or phosphatases that write disease-specific post-translational modifications (e.g., targeting Thr199 dephosphorylation during oxidative stress) offers a mechanism to selectively intercept pathological NPM1 variants. Lastly, leveraging advanced delivery systems, such as macrophage-targeted lipid nanoparticles or ischemic myocardium-homing peptides, will be crucial to restrict the intervention to the pathological microenvironment, thereby safely unlocking the therapeutic potential of NPM1 in chronic inflammatory diseases.

### 4.1 Defining the therapeutic window: acute protection vs. chronic inhibition

Implementing these precision targeting strategies requires a rigorous understanding of the spatiotemporal therapeutic window, particularly in dynamic cardiovascular events. During the acute injury phase (e.g., early hours to days post-myocardial infarction), preserving the nuclear pool of NPM1 is paramount. Therapeutic strategies during this window should strictly avoid systemic NPM1 inhibition. Instead, interventions should focus on anchoring molecules (such as HO-1 mimetics) that retain NPM1 in the nucleolus to maximize its essential DNA repair, anti-apoptotic, and cytoprotective capacities. Conversely, during the chronic remodeling phase (e.g., weeks post-infarction, or in chronic atherosclerosis and pulmonary hypertension), the therapeutic objective must pivot to targeted inhibition. Utilizing oligomerization inhibitors (e.g., NSC348884), TLR4 antagonists, or agents specifically blocking the NPM1-KDM5b interaction can effectively neutralize the cytoplasmic and extracellular pro-inflammatory actions of NPM1. This time-tailored approach breaks the vicious cycle of chronic inflammation, metabolic reprogramming, and cardiac fibrosis without abolishing the basal physiological functions of nuclear NPM1.

## 5 Conclusion

In conclusion, NPM1 is undergoing a profound conceptual evolution: from a classical, static nucleolar protein primarily studied in hematological malignancies, to a highly dynamic, context-dependent stress integrator across a broad spectrum of NCDs. By undergoing specific post-translational modifications (e.g., phosphorylation, lactylation, and S-glutathionylation) and dynamic LLPS, wild-type NPM1 senses upstream metabolic, oxidative, and genotoxic stresses, subsequently orchestrating downstream cell fate decisions through spatial relocalization and epigenetic remodeling.

On one hand, in response to acute stress, such as myocardial ischemia, NPM1 exerts a cytoprotective role in the early phase through its canonical nucleolar stress response and DNA repair chaperone functions (Avitabile et al., 2011). On the other hand, during chronic disease progression, the functions of NPM1 are often hijacked or dysregulated in context-specific ways. In AML, for example, the NPM1c + mutant utilizes its aberrant subcellular localization to hijack the Menin-KMT2A epigenetic complex, leading to abnormal expression of HOX genes and driving leukemogenesis (Salman and Stein, 2024; Brunetti et al., 2018). In cardiovascular diseases, wild-type NPM1 relies on an entirely distinct epigenetic interaction network to drive pathology. Here, NPM1 responds to chronic stress by recruiting the histone demethylase KDM5b to silence TSC1, resulting in mTOR activation and locking macrophages into a pro-inflammatory M1 phenotype (Zhang et al., 2024). Taken together, NPM1 consistently acts as a central epigenetic regulatory hub, translating upstream damage or mutational signals into profound cellular phenotypic reprogramming.

Despite these conceptual advances, fully translating NPM1 biology into clinical interventions for NCDs requires addressing several highly specific future questions. First, regarding epigenetic and PTM coding under metabolic stress: What are the specific epigenetic partners that wild-type NPM1 recruits in cardiomyocytes or neurons, and how do nutrient-driven modifications (such as K257 lactylation) dynamically alter this interactome in conditions like diabetic cardiomyopathy? Second, regarding biophysical dynamics: Can we map the exact triggers that cause the irreversible liquid-to-solid phase transition of NPM1 condensates in neurodegenerative diseases (like AD and PD) or vascular malformations, and can this process be pharmacologically reversed? Finally, regarding the immune microenvironment: Given that autoantibodies against NPM1 have shown predictive value for systemic sclerosis–associated pulmonary hypertension (Ulanet et al., 2003), how NPM1 reshapes immune homeostasis through nucleocytoplasmic shuttling or extracellular secretion as an alarmin remains a core direction urgently needing exploration.

Looking ahead, indiscriminate systemic inhibition of NPM1 may be inadvisable due to its essential physiological functions. Future therapeutic strategies must be precisely targeted. The clinical success of Menin inhibitors in NPM1c + AML elegantly demonstrates the feasibility of disrupting specific pathological NPM1 complexes without destroying basal cellular functions. Inspired by this targeted approach, a crucial question for chronic pathological processes is whether we can develop next-generation inhibitors that selectively disrupt disease-specific wild-type NPM1 interactions—such as its binding to KDM5b in macrophages or its aberrant cleavage in vascular smooth muscle cells—to promote tissue repair. In summary, NPM1 is evolving into a versatile stress integrator across a broad range of NCDs, opening up highly specific avenues for understanding and targeted intervention in these complex conditions.
